# Vasomotor menopausal disorders as a possible result of dysfunction of the microbiota-intestine-brain axis

**DOI:** 10.25122/jml-2021-0106

**Published:** 2022-02

**Authors:** Oksana Mykolaivna Pavlovska, Kateryna Mykolaivna Pavlovska, Svitlana Mykolaivna Heryak, Stefan Volodymyrovych Khmil, Mariya Stefanivna Khmil

**Affiliations:** 1. Department of Obstetrics and Gynecology, Odessa National Medical University, Odessa, Ukraine; 2. Department of Internal Medicine No.1-Cardiovascular Pathology, Odessa National Medical University, Odessa, Ukraine; 3. Second Department of Obstetrics and Gynecology, I. Horbachevsky Ternopil National Medical University, Ternopil, Ukraine; 4. First Department of Obstetrics and Gynecology, I. Horbachevsky Ternopil National Medical University, Ternopil, Ukraine

**Keywords:** menopausal vasomotor disorders, intestinal microbiota, climacteric period

## Abstract

The study involved clinical and laboratory examination of 54 women with vasomotor menopausal disorders divided into 3 subgroups depending on the duration of symptoms (not more than 12 months, about 3 years, from 5 to 7 years). The control group included 21 patients without vasomotor disorders during the menopausal period. Data from the general and obstetric-gynecological anamnesis and the results of objective and general clinical examinations were analyzed. To assess the state of intestinal microbiocenosis in patients, a bacteriological study of feces was used according to modern methods. In women with menopausal vasomotor disorders, chronic arterial hypertension, overweight, diabetes mellitus, chronic enterocolitis, and chronic pyelonephritis prevailed among somatic diseases. The study also revealed that these patients had a pronounced imbalance of the intensive microbiota, which was characterized by a significant decrease in the main representatives of the obligate microflora (Bifidobacterium, Lactobacillus) and an increase in the number of opportunistic strains (Klebsiella and Clostridiodes difficile). Considering modern concepts, a change in the microbial composition of the intestine may be one of the possible trigger factors for the occurrence of vasomotor menopausal disorders. Further research will clarify the influence of the microbiome on the formation of the pathological menopausal symptom complex and improve the preventive and therapeutic measures in this category of women.

## Introduction

The climacteric period is a physiological period in a woman’s life, corresponding to the transition from the reproductive phase to post menopause. According to the generally accepted concept, significant transformations occur in the functioning of the hypothalamic-pituitary-gonadal axis during this stage, against the background of general age-related changes in the body [1, 2]. Thus, according to the data of numerous research, involutive processes in the female reproductive system are accompanied by a progressive increase in the level of follicle-stimulating hormone (FSH), a decrease in the concentrations of estradiol (E2), anti-mullerian hormone, and inhibin B, which is caused by gradual depletion of the ovarian follicular reserve, a decrease in the frequency of the ovulatory cycles, acceleration of follicular atrophy and atresia, as well as a decrease in the expression of receptors to gonadotropins (FSH, luteinizing hormone (LH)) [3, 4]. It should be noted that melatonin, synthesized in the pineal gland, also plays an important regulatory part in the genesis of menopause. This hormone is known to be a regulator which, being extremely sensitive to structural and metabolic transformations in the reproductive system, ensures the effective functioning of the hypothalamic-pituitary-ovarian relations [5, 6].

Currently, the criteria for Stages of Reproductive Aging Workshop, STRAW+10, was improved, implemented, and successfully used by researchers and practitioners to clearly distinguish the stages of functioning of the female reproductive system [7–9]. Thus, the life of an adult woman is divided into three main periods: reproductive, menopausal transition, and postmenopause. These three long-term life cycles, in turn, are subdivided into ten stages, with the zero point being the date of the last menstrual period (stage 0). Thus, according to STRAW+10, the female reproductive period is divided into -5 (early), -4 (peak), -3b and -3a (late) stages, the transition period includes stage -2 (early) and stage -1 (late). The postmenopausal period is divided into 4 stages: +1a, +1b, +1c (early) and 2 (late). It should be noted that the period of perimenopause includes -2, -1, and + 1a stages.

The accepted staging of the female reproductive activity and the recommended diagnostic references, namely principal criteria, supportive criteria, descriptive characteristics, are based on long-term and multicenter cohort studies. It is very important to note that the characteristics of the menstrual cycle (menstrual bleeding) presented in STRAW+10, the quantitative parameters of the main biomarkers of the ovarian reserve (FSH, anti-mullerian hormone, inhibin B, antral follicle count), as well as the dominant clinical symptoms of menopause, are recommended taking into account their applicability to women with different body mass index, lifestyle and health conditions. It means that STRAW+10 is focused not only on healthy women but also on patients suffering from various chronic somatic diseases and endocrine disorders, with multi-vector direct and indirect effects on the fertility potential and hormonal balance in the body.

The following observation is of great interest: if the age of 75 years, which is the average life expectancy of a modern woman, is taken as 100%, then the duration of periods of various functional activity of the reproductive system can be expressed in approximately the following numbers: prepubertal period – 16%, reproductive – 44%, perimenopausal – 7%, postmenopausal – 33% [10–12]. That is, the menopausal period is not the end of life but the beginning of a new and, undoubtedly, important stage for a woman. In a way, it becomes a particular “exam” for the body, which gradually “tests” the adaptive potential of all organs and systems without exception in response to significant and long-term changes in metabolic balance and hormonal ratios. And it is the general well-being, cognitive, physical, and social activity of a woman during this period of her life that are the most striking indicators of spent youth and maturity.

Menopause, a natural phenomenon in a woman’s life, can sometimes acquire the features of a pathological condition that significantly disturbs the course of everyday life, psycho-emotional comfort, harmonization of interactions in the professional environment and society, that is, reducing the quality of life in general [13, 14].

From up-to-date positions, pathological menopause is characterized by an extensive range of clinical symptoms, the severity of which is very individual and variable [15]. Usually, pathological menopausal manifestations are divided into psychopathological, vasomotor, general physical, urogenital, and sexual. Likewise, menopausal disorders can be divided into early-term, the most common of which are vasomotor manifestations (hot flashes, night sweats, headaches, chills, fluctuations in blood pressure, heart palpitations), medium-term, characterized by atrophic changes in the skin and urogenital tract, and late, including such metabolic disorders as atherosclerosis and osteoporosis [16–19].

Currently, researchers and clinicians have collected evidence showing that women with menopausal psycho-emotional disorders and severe vasomotor manifestations have a highly unfavorable cardiovascular risk profile [20, 21]. Thus, the results of large-scale meta-analyses showed that there was a decrease in blood flow-mediated vascular dilatation due to endothelial dysfunction in this category of women, an increase in calcification of the large vessels, a thickening of the vascular intima with an increase in the rigidity of the vascular wall, an increase in blood viscosity, the risk of the formation of diastolic dysfunction of the left ventricle significantly increased (a marker of changes in intracardiac hemodynamics), the occurrence of atrioventricular and intraventricular conduction disorders, ventricular extrasystoles (single, such as bigeminy or trigeminy), which ultimately was accompanied by the development of isolated arterial hypertension, cardiomyopathy, coronary artery disease, and ischemic stroke compared with their peers without these manifestations [22, 23].

In most women, psycho-emotional and vasomotor manifestations occur almost simultaneously in 25% – in 3–6 months [24]. As a rule, pathological psycho-emotional symptoms develop during the menopausal transition, while vasomotor disorders more often become dominant within 12 months after menopause and last on an average up to 7 years [25].

Estrogen deficiency is not the main trigger factor in the formation of pathological menopausal manifestations. To date, the clinical diversity of the menopausal symptom complex is explained by multi-vector effects on the body, among which the key ones are genetic factors, cultural traditions, dietary characteristics, the degree of physical activity, the use of certain drugs, as well as the psycho-emotional attitude of a woman to her new life [26, 27].

It should also be emphasized that a certain part in the pathophysiology of dyshormonal disorders in the body can be played by qualitative and quantitative changes in the intestinal microbiota [28]. For example, it was found that in intestinal dysbiosis, there was a disorder in the transmission of sensory information from the intestine to the central structures of the brain (cerebral cortex) due to altered expression of the mucous membrane receptors, hypercatecholaminemia, and activation of the sympathetic part of the autonomic nervous system. This leads to increased secretion of biological substances such as serotonin, histamine, kinins etc, which in turn cause “neuro-endocrine-mediator chaos” hypothalamic-pituitary-adrenal dysfunction, affecting the visceral, sensory, and motor functions of the whole organism [29, 30]. Thus, our study aimed to identify the features of the intestinal microbiota in women with menopausal vasomotor disorders.

## Material and methods

We performed clinical and laboratory examinations of 75 women aged 49–56 years. Participants were divided into 2 groups. Group I included 54 patients complaining of vasomotor menopausal disorders (hot flashes, night sweats, headaches, chills, fluctuations in blood pressure, palpitations). Depending on the duration of the vasomotor symptoms, this group was divided into 3 subgroups:

• Ia (n=20) – duration of the vasomotor menopausal symptoms is not more than 12 months;
• Ib (n=18) – duration of the vasomotor menopausal symptoms is about 3 years;
• Ic (n=16) – duration of the vasomotor menopausal symptoms is 5 to 7 years.

Group II (control group) included 21 patients in whom the menopausal period proceeded without vasomotor disorders.

Data from general and obstetric-gynecological anamnesis were studied and thoroughly analyzed; a comprehensive objective and general clinical examination were carried out following the requirements of modern clinical protocols.

The state of intestinal microbiocenosis was assessed by bacteriological examination of feces. The content of the main representatives of obligate microflora was determined (Bifidobacterium, Lactobacillus, Escherichia coli with normal enzymatic activity, Foecal streptococci, Bacteroides) as well as facultative (opportunistic) microorganisms (pathogenic strains of E. Staphylococcus epidermidis, Candida albicans). All examined patients followed a certain diet, excluding food products that promoted fermentation in the intestine and alcohol and drugs (antibiotics) 3 days before sampling. From the moment of the last meal and the collection of stool samples, at least 10 hours passed. Samples were placed in sterile glassware and delivered to the laboratory within 2 hours. The interval between sampling of biomaterial and the beginning of culturing did not exceed 3–4 hours. A portion of feces (0.5–1.0 g) was added to a sterile, pre-weighed test tube, and after re-weighing, the weight of the sample was determined. After a series of successive dilutions, inoculations were carried out on various nutrient media. The quantitative accounting of grown microorganisms was carried out by calculating 1 g of feces, considering the dose of the inoculated material and the degree of its dilution. To process the study results, we used the method of variation statistics and nonparametric methods using the programs Excel 2000 and StatisticaforWindows v.6.0.

## Results

We performed a thorough analysis of concomitant somatic pathology in both groups (Table 1) and identified some interesting features and patterns. Arterial hypertension among patients in group I was observed 3.3 times more often than in the control group. At the same time, increased arterial pressure was most frequently observed among women in Ia (40.0%) and Ic groups (37.5%), in whom the duration of vasomotor menopausal symptoms was not more than 12 months and about 5–7 years, respectively. In patients from group Ib, increased arterial pressure was recorded in 16.7% of women, 2.2–2.4 times less often. It is quite possible that this kind of “scissors symptom” can be explained by a sharp decrease in the “protective” effect of endogenous estrogens in the mechanisms of arterial pressure regulation in women during early postmenopause (stage + 1a), followed by a period of adaptation of the cardiovascular system to new hormonal ratios in the body. An increased hypertension frequency was observed again during late postmenopause (stage +2) due to persistent and pronounced hypoestrogenism. This observation may be the basis for a furthermore in-depth study of the mechanisms of arterial pressure fluctuations in women in the perimenopausal and postmenopausal periods.

**Table 1.** Concomitant somatic diseases of patients with climacteric vasomotor disorders (subgroup Ia, Ib, Ic) and women in the control group (group II).

**Somatic diseases**	**Group I, n=54**						**Group II, n=21**	
	**Subgroup Ia, n=20**		**Subgroup Ib, n=18**		**Subgroup Ic, n=16**			
	**abs.**	**%**	**abs.**	**%**	**abs.**	**%**	**abs.**	**%**
**Arterial hypertension**	8	40.0	3	16.7	6	37.5	2	9.5
**Coronary artery disease**	-	-	-	-	1	6.3	-	-
**Iron-deficiency anemia**	5	25.0	4	22.2	3	18.8	3	14.3
**Bronchial asthma**	-	-	1	5.6	-	-	2	9.5
**Dyshormonal diseases of the mammary glands**	6	30.0	5	27.8	2	12.5	4	19.0
**Hyperthyroidism**	2	10.0	-	-	1	6.3	1	4.8
**Thyroiditis**	1	5.0	-	-	-	-	-	-
**BMI>25 kg/m^2^**	6	30.0	6	33.3	5	31.3	3	14.3
**Diabetes mellitus**	3	16.0	2	11.1	2	12.5	1	4.8
**Chronic enterocolitis**	5	25	4	22.2	5	31.3	1	4.8
**Chronic pyelonephritis**	1	5.0	-	-	4	25.0	1	4.8
**Chronic glomerulonephritis**	-	-	-	-	2	12.5	-	-
**Chronic venous insufficiency**	4	20	3	16.7	4	25	5	23.8

In addition, being overweight, diabetes mellitus, chronic enterocolitis, and chronic pyelonephritis in women with menopausal vasomotor disorders were more frequently observed than in the control group. Thus, when analyzing concomitant somatic pathology, it can be concluded that arterial hypertension, metabolic diseases such as obesity and diabetes mellitus, as well as chronic enterocolitis and urinary tract infections significantly complicate the clinical course of the perimenopause and postmenopausal period.

The next stage of our study was the investigation and comparison of the results of feces bacteriological examination in patients in the menopausal period (Table 2).

**Table 2.** The content of intestinal microorganisms in 1 g of feces in the patients with climacteric vasomotor disorders (subgroup Ia, Ib, Ic) and women in the control group (group II).

**Microorganisms**	**Group I, n=54**			**Group II, n=21**
	**Subgroup Ia, n=20**	**Subgroup Ib, n=18**	**Subgroup Ic, n=16**	
**Bifidobacterium, (×10^8^)**	29.71±6.78 (pIa-Ib=0.92) (pIa-Ic=0.013) (pIa-II=0.037)	30.55±4.84 (pIb-Ic<0.01) (pIb-II=0.021)	10.95±2.34 (pIc-II<0.01)	49.74±6.28
**Lactobacillus, (×10^6^)**	8.54±1.81 (pIa-Ib=0.777) (pIa-Ic=0.049) (pIa-II=0.030)	7.86±1.55 (pIb-Ic=0.062) (pIb-II=0.021)	4.56±0.71 (pIc-II<0.01)	21.23±5.32
**Bacteroides, (×10^8^)**	25.22±5.62 (pIa-Ib=0.801) (pIa-Ic=0.413) (pIa-II=0.761)	27.48±6.90 (pIb-Ic=0.324) (pIb-II=0.948)	19.70±3.56 (pIc-II=0.333)	28.17±7.85
**Foecal streptococci, (×10^6^)**	0.64±0.13 (pIa-Ib=0.110) (pIa-Ic=0.074) (pIa-II=0.254)	0.35±0.12 (pIb-Ic=0.583) (pIb-II=0.526)	0.23±0.18 (pIc-II=0.293)	0.45±0.10
**Enterococcus faecium, (×10^6^)**	37.24±7.64 (pIa-Ib=0.863) (pIa-Ic=0.465) (pIa-II=0.338)	39.01±6.78 (pIb-Ic=0.343) (pIb-II=0.398)	29.50±7.17 (pIc-II=0.098)	48.19±8.32
Escherichia coli with normal enzymatic activity, (×10^6^)	15.21±8.16 (pIa-Ib=0.687) (pIa-Ic=0.952) (pIa-II=0.077)	19.98±8.44 (pIb-Ic=0.602) (pIb-II=0.185)	14.60±5.76 (pIc-II=0.034)	34.91±7.14
**Escherichia coli with reduced enzymatic activity, (×10^6^)**	29.74±5.21 (pIa-Ib=0.294) (pIa-Ic=0.584) (pIa-II=0.031)	21.18±6.12 (pIb-Ic=0.708) (pIb-II=0.380)	24.78±7.29 (pIc-II=0.235)	14.50±4.36
Staphylococcus epidermidis, (×10^4^)	0.90±0.22 (pIa-Ib=0.566) (pIa-Ic=0.333) (pIa-II=0.780)	0.76±0.10 (pIb-Ic=0.109) (pIb-II=0.292)	1.25±0.28 (pIc-II=0.423)	0.98±0.18
**Klebsiella, (×10^5^)**	1.63±0.39 (pIa-Ib=0.532) (pIa-Ic=0.380) (pIa-II=0.012)	2.05±0.54 (pIb-Ic=0.858) (pIb-II=0.010)	2.18±0.48 (pIc-II=0.002)	0.57±0.09
Enterobacter, (×10^3^)	2.54±0.53 (pIa-Ib=0.760) (pIa-Ic=0.489) (pIa-II=0.824)	2.76±0.48 (pIb-Ic=0.661) (pIb-II=0.966)	3.10±0.60 (pIc-II=0.816)	2.81±1.08
**Citrobacter, (×10^3^)**	1.73±0.42 (pIa-Ib=0.594) (pIa-Ic=0.728) (pIa-II=0.700)	1.47±0.24 (pIb-Ic=0.460) (pIb-II=0.930)	2.01±0.68 (pIc-II=0.525)	1.51±0.38
Clostridiodes difficile, (×10^5^)	0.49±0.07 (pIa-Ib=0.016) (pIa-Ic=0.044) (pIa-II<0.01)	0.82±0.11 (pIb-Ic=0.219) (pIb-II<0.01)	1.34±0.40 (pIc-II<0.01)	0.19±0.04
**Proteus, (×10^3^)**	1.08±0.19 (pIa-Ib=0.192) (pIa-Ic=0.569) (pIa-II=0.562)	0.80±0.09 (pIb-Ic=0.109) (pIb-II=0.646)	1.27±0.27 (pIc-II=0.309)	0.91±0.22
Candida albicans, (×10^4^)	0.53±0.17 (pIa-Ib=0.488) (pIa-Ic=0.230) (pIa-II=0.725)	0.68±0.13 (pIb-Ic=0.463) (pIb-II=0.188)	0.87±0.22 (pIc-II=0.099)	0.46±0.10

Certain differences and patterns were identified in the composition of the intestinal microbiocenosis. For example, a significant decrease in bifidobacteria and lactobacilli was observed in women with vasomotor disorders, compared with those in whom the menopausal period had no complications. In patients from the control group (group II), these indicators were (49.74±6.28)×10^8^ and (21.23±5.32)×10^6^, respectively. In subgroup Ia, the content of Bifidobacterium was at the level of (29.71±6.78)×10^8^ (pIa-II=0.037), Lactobacillus – (8.54±1.81)×10^6^ (pIa-II=0.030), subgroup Ib – (30.55±4.84)×10^8^ (pIbII=0.021), (7.86±1.55)×10^6^ (pIb-II=0.021), respectively. In subgroup Ic, where the duration of vasomotor menopausal symptoms ranged from 5 to 7 years, had the most pronounced decrease in Bifidobacterium and Lactobacillus – (10.95±2.34)×10^8^ (pIc-II<0.01), (4.56±0.71)×10^6^ (pIc-II<0.01). In addition, the women with a complicated course of menopause had a significant increase in such opportunistic bacteria as Klebsiella and Clostridiodes difficile. So, in group II, Klebsiella indicators were within (0.57±0.09)×10^5^, Clostridiodes difficile – (0.19±0.04)×10^5^, in subgroup Ia – (1.63±0.39)×10^5^ (pIa-II=0.012), (0.49±0.07)×10^5^ (pIa-II<0.01), subgroup Ib – (2.05±0.54)×10^5^ (pIb-II=0.010), (0.82±0.11)×10^5^, (pIb-II<0.01), subgroup Ic – (2.18±0.48)×10^5^ (pIc-II=0.002) and (1.34±0.40)×10^5^ (pIc-II<0.01), respectively.

## Discussions

Our study revealed that the development of vasomotor climacteric disorders was accompanied by an imbalance of the intestinal microbiota, which was characterized by a significant decrease in the main representatives of the obligate microflora (Bifidobacterium, Lactobacillus) and an increase in the number of strains of such opportunistic microorganisms as Klebsiella and Clostridiodes difficile. It is important to emphasize that the most frequent cause of an increase in the proportion of these opportunistic bacteria in the intestinal microbiota is antibiotic therapy with clindamycin, third generation cephalosporins, penicillins, and fluoroquinolones [31, 32]. Therefore, it is possible to assume that women suffering from chronic infectious diseases may be at risk of forming a pathological menopausal symptom complex. This dependence was shown in our study. In turn, overgrowth of Klebsiella and Clostridiodes difficile can cause the development of antibiotic-associated pseudomembranous colitis [33, 34].

At present, many researchers identified some mechanisms of interaction of the central, peripheral nervous system, and gastrointestinal tract in their clinical and experimental work. The gastrointestinal tract, inhabited by a large group (about 1000) of various bacteria in a symbiotic relationship, creates a unique and highly individual biosphere in the body [35]. Microbial populations in the intestine synthesize folic acid, vitamin K2 (Menaquinone), vitamins B1 (Thiaminpyrophosphate), B2 (Flavinadenine dinucleotide, Flavin mononucleotide), B3 (Nicotinic acid, Nicotinamide), B5 (Free pantothenic acid), B6 (Pyridoxal phosphate), B7 (Free Biotin), B9 (Tetrahydrofolate), B12 (Cyanocobalamin), metabolites of short-chain fatty acids (acetate, propionate, and butyrate), stimulate the absorption of calcium, magnesium, iron, vitamin D [36, 37]. That is, the intestine also performs a significant immuno-hormonal function in the body except for hydrolysis of food products, absorption of substrates, excretory and detoxification function, which in current thinking is carried out and regulated by interactive communication between the vagus and enteric nervous system – the largest and most difficult part of the peripheral nervous system, which is recently actively studied [38]. The most important components of the enteric nervous system are plexus myentericus (Auerbach’s plexus), plexus submucosal (Meissner’s plexus), plexus subserous, enteric glial cells (EGCs), and Enterochromaffin cells (EC cells), which form a kind of collector of afferent information from internal organs to the central nervous system [39]. This interaction is carried out using more than 30 neurotransmitters known to date, endocrine mediators, metabolites (short-chain fatty acids), food amino acids etc [40, 41]. Therefore, according to modern scientific views and microbiota theories, the vagus nerve and the enteric nervous system are fundamental and equally important components of the intestine-brain axis, which plays a significant part in the immune, hormonal and neuroendocrine status of the body throughout life [42, 43]. Thus, a change in the microbial composition of the intestine can play an important part in the pathophysiology of the menopausal period. These aspects open a new page in the study of possible risk factors and trigger points in menopausal disorders and complications. Conducting research in this area with more strict sampling will clarify the direction of such changes in the microbiome, and it is quite possible that these may radically change the vector of diagnostic search, and accordingly, preventive and therapeutic measures.

## Conclusions

Today, the formation of the pathological menopausal symptom complex can be viewed from the standpoint of dysfunction of the axis “Microbiota-intestine-brain”. The study investigated that imbalance of the intestinal microbiota, which was characterized by a significant decrease in the main representatives of the obligate microflora (Bifidobacterium, Lactobacillus) and an increase in the number of conditionally pathogenic micro-organisms strains (Klebsiella and Clostridiodes difficile), could be one of the possible trigger factors for the occurrence of vasomotor menopausal disorders.

## Acknowledgments

### Conflict of Interest

The authors confirm that there are no conflicts of interest.

### Ethical approval

The study was approved by the Meeting of the Bioethics Commission of I. Horbachevsky Ternopil National Medical University of the Ministry of Health of Ukraine (excerpt from protocol No. 66/November 01/2021).

### Consent to participate

Written informed consent was obtained from the participants in the study.

### Data availability

The data of this study is available by request.

### Authorship

OM was responsible for the clinical management of the patients and the drafting of the manuscript. KM contributed to the methodology. SM contributed to conceptualizing. SV contributed to editing the manuscript. MS contributed to the data analysis and conducted the statistical analysis.
